# Filamentous Fungi Producing l-Asparaginase with Low Glutaminase Activity Isolated from Brazilian Savanna Soil

**DOI:** 10.3390/pharmaceutics13081268

**Published:** 2021-08-17

**Authors:** Marcela Freitas, Paula Souza, Samuel Cardoso, Kellen Cruvinel, Letícia Santos Abrunhosa, Edivaldo X. Ferreira Filho, João Inácio, Danilo Batista Pinho, Adalberto Pessoa, Pérola O. Magalhães

**Affiliations:** 1 Health Sciences School, University of Brasília, Brasília 70910-900, Brazil; marcelamdf1@gmail.com (M.F.); paulasouza@unb.br (P.S.); samuel.cardoso@aluno.unb.br (S.C.); kellen.andrade@aluno.unb.br (K.C.); leticiasaab@gmail.com (L.S.A.); 2 Institute of Biological Sciences, University of Brasília, Brasília 70910-900, Brazil; eximenes@unb.br (E.X.F.F.); danilopinho@unb.br (D.B.P.); 3 School of Pharmacy and Biomolecular Sciences, University of Brighton, Brighton BN2 4GJ, UK; J.Inacio@brighton.ac.uk; 4 Department of Biochemical and Pharmaceutical Technology, University of São Paulo, São Paulo 05508-000, Brazil; pessoajr@usp.br

**Keywords:** l-asparaginase, filamentous fungi, *Penicillium*, *Fusarium fujikuroi* species complex, acute lymphoblastic leukemia, Plackett–Burman design

## Abstract

l-asparaginase is an enzyme used as treatment for acute lymphoblastic leukemia (ALL) due to its ability to hydrolyze l-asparagine, an essential amino acid synthesized by normal cells unlike neoplastic cells. The adverse effects of l-asparaginase formulations are associated with its glutaminase activity and bacterial origin; therefore, it is important to find new sources of l-asparaginase-producing eukaryotic microorganisms with low glutaminase activity. This work evaluated the biotechnological potential of filamentous fungi isolated from Brazilian Savanna soil and plants for l-asparaginase production. Thirty-nine isolates were screened for enzyme production using the plate assay, followed by measuring enzymatic activity in cells after submerged fermentation. The variables influencing l-asparaginase production were evaluated using Plackett–Burman design. Cell disruption methods were evaluated for l-asparaginase release. *Penicillium sizovae* 2DSST1 and *Fusarium proliferatum* DCFS10 showed the highest l-asparaginase activity levels and the lowest glutaminase activity levels. *Penicillium sizovae* l-asparaginase was repressed by carbon sources, whereas higher carbon concentrations enhanced l-asparaginase by *F. proliferatum*. Maximum enzyme productivity, specific enzyme yield and the biomass conversion factor in the enzyme increased after Plackett–Burman design. Freeze-grinding released 5-fold more l-asparaginase from cells than sonication. This study shows two species, which have not yet been reported, as sources of l-asparaginase with possible reduced immunogenicity for ALL therapy.

## 1. Introduction

The leading cause of death from disease among children is cancer, with leukemias being the most common type diagnosed in children up to 14 years of age, mainly acute lymphoblastic leukemia (ALL). l-asparaginase (EC 3.5.1.1; l-asparagine amidohydrolase), listed by the World Health Organization as an essential medicine, is an important enzyme in the pharmaceutical field that has been used for ALL treatment in children since 1970 [1,2]. Its anti-leukemic effect relies on the hydrolysis of l-asparagine, an amino acid required for the biosynthesis of proteins that neoplastic cells cannot synthesize due to lack of l-asparagine synthetase, unlike normal cells. l-asparaginase and asparagine synthetase can also use glutamine as substrate: l-asparaginase cleaves its amino group off at a slower rate and asparagine synthetase uses glutamine to provide the amine and connects to aspartate, thus forming asparagine [3,4]. Lang first reported l-asparaginase activity in 1904, followed by Furth and Friedmann in 1910 and Clementi in 1922 [5]. In 1953, Kidd described the regression of transplanted lymphomas induced in vivo in mouse and rat cells by guinea pig serum, which was later attributed to its l-asparaginase activity [6]. However, the need for large-scale production and purification of this enzyme for therapeutic work from guinea pigs was difficult, which led to a search for alternative sources with similar anti-leukemic effects. l-asparaginase is widely distributed in nature, being found in plants, microorganisms, and tissues of several animals such as fishes, mammals, and birds. Although enzyme properties and tumor inhibitory activity differ between organisms, microorganisms such as fungi and bacteria were found to be efficient and inexpensive sources of l-asparaginase due to large-scale production and their ability to grow easily on simple and inexpensive substrates [2,7,8].

l-asparaginase production is divided into upstream and downstream processing, where the upstream process development includes the selection of the cell line, culture media, selection of the cultivation process, bioreactor parameters such as pH and temperature, and optimization, while downstream processing includes all the steps required for enzyme purification [9]. Commercialized l-asparaginase formulations include native and PEGylated asparaginases from *Escherichia coli* and a native asparaginase purified from *Erwinia carotovora* [10]. However, adverse effects have been reported when *E. coli* asparaginase is administered, ranging from mild allergic reactions to anaphylactic shock in approximately 5–50% of the patients treated in various clinical trials [11]. *Erwinia* asparaginase, with similar antitumor activity but of different antigenic structure, is administered in responding but antigenically sensitive patients as an alternative to *E. coli* asparaginase [8]. However, allergic reactions in children with leukemia and lymphoma and venous thrombotic complications in adults undergoing induction treatment for ALL have been reported when native asparaginases from *E. coli* and *Erwinia* were administered [12,13]. Additionally, the formation of anti-*Erwinia* asparaginase antibodies and subsequently altered pharmacokinetics of *Erwinia* asparaginase during intravenous and intramuscular therapy were reported [14]. Although the biological half-life of asparaginase is extended through the PEGylation process, plasmatic asparagine and glutamine are hydrolyzed into ammonia, which may be responsible for the high incidence of symptomatic hyperammonemia in children with ALL receiving PEG-asparaginase [15]. An immune response, given partly by the protein’s large size and bacterial origin, is promoted by cleavage of l-asparaginase by normal and leukemic lymphoblasts through lysosomal proteases, thus potentiating antigen processing, which is the main reason for the interruption of l-asparaginase treatment [16]. In this scenario, an enzyme that leads to fewer side effects could be found in other microbial sources such as eukaryotic microorganisms, which present potentially better compatibility with the human immune system due to their evolutionary proximity [17,18]. Another factor that contributes to asparaginase-associated toxic side effects is its glutaminase activity [19]. Additionally, certain cells of the body may be sensitive to a deficiency of glutamine as an action of glutaminase [20]. Among the reported asparaginase formulations, there are asparaginases with undetected glutaminase activity, others with low to moderate activity, and some others with augmented glutaminase activities. Of the three asparaginases licensed by the US Food and Drug Administration, all of which are fermentation products, *E. coli* asparaginases have relatively low glutaminase activity, while *Erwinia* asparaginase has a higher glutaminase moiety, approximately 10-fold higher than that of *E. coli* and, therefore, K_M_ and V_MAX_ more favorable for deamination of glutamine [21].

In addition to its use as an antitumor drug, application of asparaginase in the food industry offers a possible alternative method for mitigating acrylamide that should have a limiting effect on the overall formation of Maillard products. Non-enzymatic browning generally occurs at temperatures above 100 °C and is responsible for the color and flavor development of fried and cooked foods. The predominant route for acrylamide formation, a carcinogen, is through the Maillard reaction between asparagine and reducing sugars. Asparaginase can reduce the level of free asparagine by specifically removing one of the essential acrylamide precursors [22]. Commercially, there are two preparations of asparaginase derived from fungi used by the food industry: PreventASe^®^ is a recombinant form of the enzyme derived from *Aspergillus niger* and Acrylaway^®^ is the trade name of a native asparaginase obtained from *Aspergillus oryzae* [23].

The Brazilian Savanna, also known as Cerrado, is a global biodiversity hotspot, with over 4800 plant and vertebrate species found nowhere else [24,25]. Located in the center of Brazil with outliers in São Paulo and the Northeast, the continuous Brazilian Savanna region is made up of a core area comprising almost all of Goiás, western Bahia, western Minas Gerais, and all of eastern Mato Grosso, with its geographical center near the city of Goiás Velho, west of Brasília. The Brazilian Savanna has tropical vegetation, with an average annual temperature of 20–26 °C. Extreme temperatures vary from 14 °C to 44 °C in southern São Paulo. The Cerrado climate is characterized by a rainy season followed by a dry season. The average rainfall per year ranges from 750 to 2000 mm (in São Paulo 1000–1500 mm), and the average rainfall of the driest month (July or August) during the dry season ranges from 5 to 40 mm with an average of 10–30 mm [26]. An appealing advancement in therapeutic applications with fewer side effects is to explore microorganisms isolated from extreme environments [27,28,29,30,31]. Therefore, we investigate whether filamentous fungi adapted to Brazilian Savanna climate conditions isolated from the soil and plants of the Goiás (GO) and Distrito Federal (DF) regions possess a potential biotechnological application in l-asparaginase production of pharmaceutical interest with low glutaminase activity, thus potentially less immunogenic, and optimize the upstream processing for enhanced l-asparaginase production of selected fungi.

## 2. Materials and Methods

### 2.1. Fungal Strains and Maintenance

Soil samples were collected in Brasília—DF and Goiás—GO, Brazil (Savanna region) in sterilized flasks containing 10 mL of sterile saline solution. The collected soil was subjected to serial dilution in sterile saline and an aliquot of each soil dilution was deposited onto potato dextrose agar (PDA) supplemented with 0.1% (*w/v*) ampicillin and incubated at 28 °C. The strains isolated from the Brazilian Savanna soil were deposited within the scope of the SisBiota Brasil (National System of Research in Biodiversity—CNPq) of filamentous fungi with authorization to access or send a sample of the genetic heritage component number 010770/2013-5 and access authorization by the National Genetic Heritage Management System and the Traditional Knowledge Associated Genetic Heritage Management Council in compliance with the provisions of Law No. 13,123/2015 and its regulations (registration number: AEFBB51 Pérola de Oliveira Magalhães Dias Batista). The strains are maintained by the Laboratory of Enzymology of the Institute of Biological Sciences at University of Brasília, Brazil, and were donated by Professor Edivaldo Ximenes Ferreira Filho.

Leaves of plants grown in the Brazilian Savanna were harvested in Darcy Ribeiro Campus of the University of Brasília, Brazil, and surroundings. The endophytic fungi were isolated [32] and grown on Sabouraud dextrose agar with chloramphenicol and 2% (*w/v*) malt extract and incubated at 28 °C. All soil and endophytic isolates were maintained by transfer of mycelial discs (agar plugs of 8 mm in diameter) onto fresh plates and incubated at 28 °C. Isolated *A. terreus* 2DCSS6 was used as a positive control in l-asparaginase screening assays [27].

### 2.2. Culture Media

Growth induction culture medium: 2.4% (*w/v*) potato dextrose broth and 1% (*w/v*) yeast extract

MCDM 1: 0.2% (*w/v*) glucose, 1.0% (*w/v*) l-asparagine, 0.152% (*w/v*) KH_2_PO_4_, 0.052% (*w/v*) KCl, 0.052% (*w/v*) MgSO_4_·7H_2_O, trace amounts of CuNO_3_·3H_2_O, ZnSO_4_·7H_2_O and FeSO_4_·7H_2_O and 2% (*w/v*) agar. The medium was supplemented with 0.009% (*w/v*) stock solution of 2.5% (*w/v*) phenol red prepared in ethanol and the final pH of the medium was adjusted to 6.2.

MCDM 2: 1.71% (*w/v*) l-proline, 1.99% (*w/v*) NaNO_3_, 1.38% (*w/v*) l-asparagine, 0.65% (*w/v*) glucose, 0.0152% (*w/v*) K_2_HPO_4_, 0.052% (*w/v*) MgSO_4_·7H_2_O, 0.052% (*w/v*) KCl, 0.001% (*w/v*) ZnSO_4_·7H_2_O, 0.001% (*w/v*) FeSO_4_·7H_2_O, 0.001% (*w/v*) CuSO_4_·5H_2_O, pH adjusted to 6.5 with 5 M KOH.

MCDM 3: growth induction medium supplemented with MCDM2.

MCDM 4: 0.152% (*w/v*) K_2_HPO_4_; 0.052% (*w/v*) KCl; 0.052% (*w/v*) MgSO_4_·7H_2_O; 0.001% (*w/v*) ZnSO_4_·7H_2_O; 0.001% (*w/v*) FeSO_4_·7H_2_O and 0.001% (*w/v*) CuSO_4_·5H_2_O.

### 2.3. Semi-Quantitative l-Asparaginase Screening

Semi-quantitative screening of l-asparaginase-producing microorganisms was carried out by solid-state method using modified Czapek Dox medium (MCDM) 1 [33]. Control plates were prepared as described above, replacing l-asparagine with sodium nitrate as nitrogen source. The media were autoclaved and poured into plates (20 mL). One mycelial disc of each isolate was inoculated onto l-asparaginase-screening and control plates and incubated at 28 °C for 48 h. The colony diameter and zone diameter were determined by measuring the inner and outer diameter of the microorganisms’ growth and enzyme production, respectively. Zone index was calculated as the ratio of outer to inner diameter as shown in Equation (1) [27].

(1) $$\mathrm{Zone}\;\mathrm{Index}=\frac{\mathrm{Zone}\;\mathrm{diameter}}{\mathrm{Colony}\;\mathrm{diameter}}$$

### 2.4. Quantitative l-Asparaginase Screening

Quantitative screening of l-asparaginase-producing microorganisms was carried out by measuring enzyme activity in fungal cells. All submerged fermentation experiments were carried out in autoclaved 250 mL Erlenmeyer flasks containing 50 mL of culture medium, in duplicate. One mycelial disc of each isolate was inoculated into a growth induction culture medium incubated at 30 °C and 120 rpm for 72 h. The culture media were filtered, and the biomass was transferred to MCDM2 [34]. The flasks were incubated at 30 °C and 120 rpm for 120 h. The culture media were filtered, and the biomass was harvested.

### 2.5. Determination of l-Asparaginase Activity

Enzyme assay was performed by measuring the l-asparaginase activity in the cells from the amount of aspartate hydroxamate produced from asparagine and hydroxylamine [35]. The reaction mixture consists of 0.1 g fungal cells, 1.5 mL 50 mM Tris-HCl buffer pH 8.6, 0.2 mL 100 mM stock l-asparagine solution (final concentration 10 mM) and 0.2 mL 1 M stock hydroxylamine solution (final concentration 100 mM) previously neutralized with 2 M NaOH (total volume 2 mL). The reaction mixture was incubated at 37 °C and 200 rpm. After 30 min, 0.5 mL ferric chloride reagent (10% (*w/v*) FeCl_3_ and 20% (*w/v*) trichloroacetic acid in 0.66 M HCl) was added. Sample blank was performed with incubation of the buffer and the sample under the same conditions described above, followed by addition of l-asparagine and hydroxylamine stock solutions after ferric chloride reagent. The samples were centrifuged at 9000 rpm for 5 min at 4 °C. The absorbance of the supernatant was measured in a spectrophotometer at 500 nm. A l-aspartic acid β-hydroxamate standard curve (0–3.0 µmol) was constructed with 50 mM Tris-HCl buffer pH 8.6 and 0.5 mL ferric chloride reagent, in triplicate. The obtained equation was y = 0.3029x + 0.0017, R^2^ = 0.9998, where y is the absorbance and x is the l-aspartic acid β-hydroxamate formed in µmol. For all cell analyses, each isolate was evaluated with two biological repetitions performed in analytical duplicates (*n* = 4) and the results are presented as mean ± standard deviation of enzyme activity. One unit of l-asparaginase was defined as the amount of enzyme needed to form 1 µmol of l-aspartic acid β-hydroxamate per minute per gram of cell as shown in Equation (2).

(2) $$\mathrm{Enzyme}\;\mathrm{activity}\;\left({U\;g}^{-1}\right)=\frac{\left(\text{µ}\mathrm{mol}\;\mathrm{of}\;L\;-\;\mathrm{aspartic}\;\mathrm{acid}\;\beta -\mathrm{hydroxamate}\;\mathrm{formed}\right)}{\left(\min\mathrm{ofincubation}\times g\;\mathrm{of}\;\mathrm{cells}\;\mathrm{taken}\right)}$$

### 2.6. Determination of l-Glutaminase Activity

The isolates that presented the largest l-asparaginase halos on the plate assay and the highest l-asparaginase activity values were further screened for glutaminase activity under submerged fermentation. One mycelial disc of selected isolates was inoculated into MCDM3 incubated at 30 °C and 120 rpm for 120 h. Glutaminase activity was quantified as described for l-asparaginase by replacing l-asparagine for glutamine as the reaction substrate. One unit of glutaminase was defined as the amount of enzyme needed to form 1 µmol of glutamic acid γ-hydroxamate per minute per gram of cells. Statistical significance was determined using unpaired *t*-test, with alpha = 0.05. Each fungal isolate was analyzed individually using GraphPad Prism software v.5.01 to evaluate significant differences between l-asparaginase and glutaminase activity levels.

### 2.7. Identification of the Fungal Species

#### 2.7.1. DNA Extraction and PCR Amplification

Genomic DNA extraction was performed using Wizard Genomic DNA Purification Kit (Promega Corporation, Madison, WI, USA) from mycelia grown on PDA, incubated at 28 °C for 7 days. DNA concentration and quality were determined using Nanodrop ND-1000 spectrometer (Thermo Scientific, Waltham, MA, USA) and preparations were adjusted to 20 ng/µL of the DNA template.

Amplification reactions were performed with the primers for each gene using GoTaq^®^ DNA Polymerase (Promega Corporation, Madison, WI, USA) according to the manufacturer’s instructions. The 3’ end of the 18rDNA, ITS1, 5.8S rDNA, ITS2 and the 5’ end of the 28rDNA (*its*) were amplified using the primer pair ITS1-F (5’-CTTGGTCATTTAGAGGAAGTAA-3’)/ITS-4 (5’-TCCTCCGCTTATTGATATGC-3’) [36,37]. PCR amplifications of β-tubulin (*tub*2), elongation factor-1 alpha (*ef*-1*α*), calmodulin (*cam*) and RNA polymerase II subunit (*rpb*2) were performed using the following primer pairs: Bt2a (5’-GGTAACCAAATCGGTGCTGCTTTC-3’)/Bt2b (5’-ACCCTCAGTGTAGTGACCCTTGGC-3’) [38]; EF1-728F (5’-CATCGAGAAGTTCGAGAAGG-3’)/EF1-986R (5’-TACTTGAAGGAACCCTTACC-3’) [39]; cmd5 (5′-CCGAGTACAAGGAGGCCTTC-3′)/cmd6 (5′-CCGATAGAGGTCATAACGTGG-3′) [40]; and fRPB2-5F (5′-GA(T/C)GA(T/C)(A/C)G(A/T)GATCA(T/C)TT(T/C)GG-3′)/fRPB2-7cR (5′-CCCAT(A/G)GCTTG(T/C)TT(A/G)CCCAT-3′) [41], respectively.

PCRs were performed using the recommended regions for each genus, i.e., *tub*2, *ef*-1*α*, and *its* for *Fusarium* [42,43] and *tub*2, *cam*, *its* and *rpb*2 for *Penicillium* [44]. PCR was performed with an initial denaturation of 95 °C for 4 min, followed by 35 cycles of 95 °C for 30 s (*its* and *ef*-1*α*) or 94 °C for 1 min (*rpb*2, *tub*2 and *cam*), annealing at 55 °C (*its*), 58 °C (*ef*-1*α*) for 30 s, 47 °C (*rpb*2), 58 °C (*tub*2) or 50 °C (*cam*) for 1 min, initial extension at 72 °C for 30 s (*its*) or 1 min and at 72 °C for 10 min on the final extension. PCR amplicons were analyzed by 1.0% agarose gel electrophoresis along with a DNA molecular weight marker (GeneRuler 1kb gene, Thermo Fisher Scientific, Waltham, MA, USA). The PCR products were purified using a Pure Link PCR purification kit (Thermo Scientific) and sequencing was performed directly from the purified PCR-amplified fragments using the automated sequencer MegaBACE 500™.

#### 2.7.2. DNA Sequencing and Phylogenetic Analysis

The nucleotide sequence datasets were constructed using *tub*2 and *ef*-1*α* for *Fusarium* [42,43], or *tub*2, *cam*, *its* and *rpb*2 for *Penicillium* [44,45] from the two isolates characterized here, and representative isolates of the *Fusarium* or *Penicillium.* To test possible topological incongruences, phylogenetic trees were individually obtained from each genomic region. Multiple alignments were obtained with MAFFT v7. Finally, phylogenetic trees were reconstructed, for the concatenate data (*its*, *cam*, *tub2*, *rpb*2 for *Penicillium* or *tub*2 and *ef-*1*α* for *Fusarium*), using Bayesian inference (BI). The best substitution models for each partition were determined with MrModeltest [46]. The CIPRES web portal [47] was used to run MrBayes v3.2.1 [48]. The Markov chain Monte Carlo (MCMC) analysis was run with a total of 10 million generations, sampling every 1000 generations. The convergence of the log likelihoods was confirmed using TRACER v1.7.1 [49]. The first 25% of the sampled trees were discarded as burn-in, with the posterior probability (PP) values calculated with the remaining trees [50]. The phylogenetic tree was edited in FigTree v1.4 [49] and Inkscape (www.inkscape.org, accessed on 3 July 2020).

### 2.8. Screening of Nutrient and Culture Conditions by Plackett–Burman Design

Twelve independent variables previously evaluated in the optimization of the culture medium for the production of l-asparaginase by filamentous fungi [34,51,52] were selected to evaluate their effects on l-asparaginase activity and specific enzyme activity by Plackett–Burman design (PBD) at two levels (Table 1).

The PBD matrix and the data analysis were determined using the software Protimiza Experimental Design with 16 combinations of the 12 variables to be evaluated of one genuine repetition each, with triplicates of the central point, totaling 19 runs. The isolates that presented the highest levels of l-asparaginase activity with the lowest values of glutaminase activity were inoculated into MCDM4 combined with variables according to the PBD matrix (Table 2), cultivated at 120 rpm for 48 h (*Penicillium* sp. 2DSST1) and 96 h (*Fusarium* sp. DCFS10).

The culture media were filtered, and the biomass was harvested, washed with distilled water, weighed, and stored at −80 °C. Enzyme extraction was carried out with frozen cells ground in a pre-chilled mortar and pestle in an ice bath until a powder was obtained. The macerated biomass was suspended (0.5 g mL^−1^) in 50 mM Tris-HCl buffer pH 8.6 and homogenized on a vortex for approximately one minute. The homogenate was centrifuged at 4000× *g* for 5 min at 4 °C and the supernatant was used as a crude enzyme extract. l-asparaginase activity assay was performed as described previously with a crude enzyme extract (0.1 mL) in place of cells. Therefore, one unit of l-asparaginase was defined as the amount of enzyme needed to form 1 µmol of l-aspartic acid β-hydroxamate per minute per mL of crude extract. All crude extract analyses were performed in triplicate within each biological repetition (*n* = 3) and the results are presented as mean of enzyme activities with standard deviation for each isolate.

### 2.9. Quantification of Total Protein

Protein quantitation of samples submitted to the microbial cell disruption method was determined with Pierce BCA Protein Assay Kit (Thermo Scientific, Waltham, MA, USA) to calculate the specific enzyme activity (U mg^−1^). In total, 200 µL of a freshly prepared reagent was added to a 25 µL aliquot of the supernatant of the disrupted cell sample, in triplicate, into a 96-well plate and incubated at 37 °C for 30 min. The absorbance of the reaction was measured on a spectrophotometer at 562 nm. A bovine serum albumin standard curve (0–2.0 mg mL^−1^) was constructed with 50 mM Tris-HCl buffer pH 8.6, in triplicate, to determine the total protein concentration in samples.

### 2.10. Kinetic Parameters of Cellular Growth and l-Asparaginase Activity

The kinetic parameters of cellular growth and l-asparaginase activity produced by the selected isolates were evaluated before and after the screening of culture media variables. A mycelial disc of the selected isolates was inoculated into MCDM3 as described previously and incubated at 30 °C and 120 rpm for 120 h. The selected isolates were cultivated in the culture medium in which the highest l-asparaginase activity value was obtained according to PBD, incubated at 32 °C and 120 rpm for 120 h. The cells were harvested every 24 h, weighed, the enzyme was extracted, and l-asparaginase activity was determined. The l-asparaginase specific activity was calculated and plotted until biomass started to decrease, indicating cell death. The maximum biomass productivity (P_X,max_), maximum enzyme productivity (P_E,max_), specific growth rate (µ_max_), specific enzyme productivity (μ_E,max_) and biomass conversion factor in enzyme (Y_E/X_) were calculated according to Equations (3)–(7), respectively, where X is the biomass concentration (g L^−1^), E is the enzymatic activity (U L^−1^) and t is the time (h).

(3) $$P_{X,\max}=\frac{\left(X_{1}-{\;X}_{0}\right)}{\left(t_{1}-{\;t}_{0}\right)}$$

(4) $$P_{E,\max}=\frac{\left(E_{1}-{\;E}_{0}\right)}{\left(t_{1}-{\;t}_{0}\right)}$$

(5) $$µ=\frac{\ln\left(\frac{X_{1}}{X_{0}}\right)}{\left(t_{1}-{\;t}_{0}\right)}$$

(6) $${\text{µ}}_{E,\max}=\frac{E}{X\;\times t}$$

(7) $$Y_{\frac{E}{X}}=\frac{E}{X}$$

### 2.11. Fungal Cell Disruption Mechanical Methods for l-Asparaginase Release

Freeze-grinding and sonication of fungal cells were compared to evaluate which method releases more l-asparaginase from the cells. The enzyme was extracted by the freeze-grinding method as described previously, with frozen cells ground in a pre-chilled mortar and pestle in an ice bath until a powder was obtained. The macerated biomass was suspended in Tris-HCl buffer, homogenized on a vortex, and centrifuged and the supernatant was used as a crude enzyme extract. For sonication, thawed cells were suspended (0.5 g mL^−1^) in 50 mM Tris-HCl buffer pH 8.6 and sonicated in a Model 120 Sonic Dismembrator (model FB-120, Thermo Fisher Scientific). The distance between the bottom of the 50 mL centrifuge tube where the sample was stored and the sonicator tip end was maintained at approximately 1 cm throughout the process. Sonication was performed at 120 W, 20 kHz and 40% amplitude in an ice bath. The cells were sonicated for 8 cycles of 59 s pulse on and then allowed to cool down for 30 s pulse off [53]. The homogenate was centrifuged at 3100× *g* for 15 min at 4 °C and the supernatant was used as crude enzyme extract.

### 2.12. Scanning Electron Microscopy Analysis

Morphological changes of *Penicillium* sp. 2DSST1 subjected to mechanical methods for fungal cell disruption were evaluated under scanning electron microscopy (SEM). Biomass that had previously been macerated, sonicated and untreated, used as a control, were prepared as liquid cultured microorganisms [54]. Cells (100 mg) were resuspended in a fixative buffer (0.1 M sodium cacodylate with 5% (*w/v*) glutaraldehyde, pH 7.2), followed by resuspension in sodium cacodylate buffer without glutaraldehyde. Samples were washed with autoclaved water, dehydrated through increased ethanol concentrations (35, 50, 75, 95 and 100%) and air dried with hexamethyldisilazane for 15 min twice [55]. Samples were mounted onto a SEM sample stub with a carbon adhesive tape, sputter coated with 4 nm platinum using a Quorum Technologies Q150T sputter coater (Quorum Technologies Ltd., Lewis, UK) and imaged with a Zeiss SIGMA field emission gun scanning electron microscope (FEG-SEM, Carl Zeiss AG, Jena, Germany) using a Zeiss Everhart-Thornley secondary electron detector with electron high tension at 5.00 kV, signal A SE2, and working distance of 8.5 mm.

## 3. Results

Thirty-nine fungal isolates from the Brazilian Savanna were evaluated for l-asparaginase production, out of which 21 were isolated from the soil and 18 from plants.

### 3.1. Primary Screening Using the Semi-Quantitative Method

**Figure 1:**
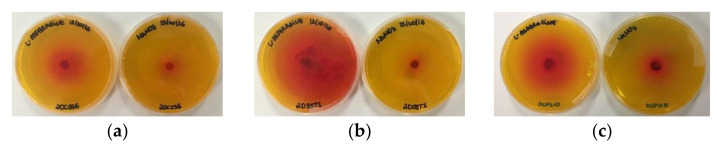
Semi-quantitative screening of the l-asparaginase production of fungi isolated from the Brazilian Savanna in a solid medium supplemented with phenol red containing a single source of nitrogen (l-asparagine or sodium nitrate). The presence of a red halo on the plates represents the potential of high l-asparaginase production. l-asparagine as enzyme substrate (left) and sodium nitrate as control (right): (**a**) 2DCSS6; (**b**) 2DSST1; (**c**) DCFS10.

l-asparaginase production is accompanied by an increase in the pH of the culture filtrates due to the breakdown of l-asparagine releasing aspartic acid and ammonia, which alkalinizes the medium. The plate assay utilizes this principle by incorporating the pH indicator phenol red in a medium containing l-asparagine, which turns pink at alkaline pH; thus, a pink zone is formed around microbial colonies producing l-asparaginase (Figure 1) [33]. 

Out of the 39 isolates from the Brazilian Savanna soil and plants, 27 showed growth on l-asparagine media after 48 h of incubation, of which 14 (14/27) isolates showed a zone diameter twice the colony diameter (index > 2): two *Aspergillus* isolates (DCFS1 and 2DCSS6), eight *Penicillium* isolates (DCFS6, RCFS24, 2DSST1, 2DMGSE2, RCFS6, 2DSST10, RCFT14 and DCFF2), one *Fusarium* isolate (DCFS10) and three unidentified endophytes (CAG2, EP03 and EP01). Twelve isolates (12/39) did not grow on l-asparagine media, out of which 10 isolates were endophytes (BR, CAG, CAG1, CAG3, CAM01, CB02, GOI03, IPE02, OH01, OH03, *Aspergillus* sp. DCFS9 and *Fusarium* sp. RCFS3). Results are given as zone index values, calculated as the ratio of the zone diameter to the colony diameter, as shown in Table 3.

The isolates of fungi from the Brazilian Savanna showed higher or similar zone indexes in a shorter incubation period compared to the data found in the literature. The maximum value of the zone index for *Trichosporon asahii* IBBLA1 isolated from Antarctica was 5.8 after 96 h of incubation [27]. *Curvularia* sp. S3.4, *Rhizopus* sp. W3, *Rhizopus* sp. W5, *Aspergillus* sp. C3, *Aspergillus* sp. C7 and *Aspergillus* sp. MTCC 1782 isolated from the soils of India produced zone indices ranging from 1.0 to 2.40 and 1.18 to 2.40 using phenol red and bromothymol blue indicators, respectively, after 72 h of incubation [18]. *Penicillium* sp. T6.2, *Penicillium* sp. T8.3, and *Fusarium* sp. T22.2 produced a zone index greater than 1.0 after 72 h in cultures inoculated with conidia; *Penicillium* sp. T9.1 also produced a zone index greater than 1.0, but after 168 h, while *Penicillium* sp. T6.1 reached zone index 0.88 after 168 h using bromothymol blue dye [56].

### 3.2. Confirmatory Screening Using Quantitative Method

**Figure 2:**
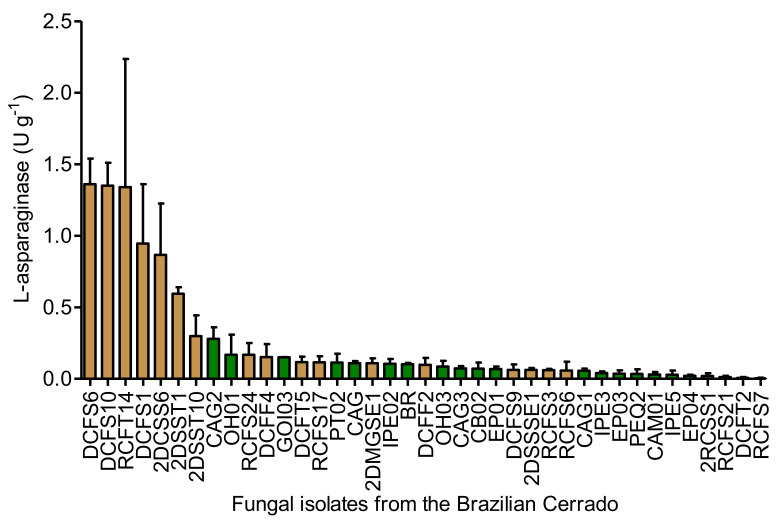
Distribution of l-asparaginase activity quantified in the cells of 39 fungal isolates from the Brazilian Savanna grown under submerged fermentation. Results are presented as mean ± standard deviation of enzyme activity. Brown bar: soil isolates; green bar: endophytes.

The quantitative assay for the determination of l-asparaginase-producing fungi was performed after submerged fermentation of each isolate. l-asparaginase activity was measured in the cells from the amount of aspartate hydroxamate produced from asparagine and hydroxylamine (Figure 2), a method considered the most sensitive when compared to ammonia quantitation produced from asparagine using Nessler’s reagent, although giving similar results [57]. However, current estimates of l-asparaginase activity reported in the literature may be overestimated when Nessler reagent is used. The Nessler method is an indirect measurement of asparaginase activity that determines the concentration of ammonia; however, ammonia is also generated throughout microbial fermentations, which will also reduce the Nessler reagent if crude microbial extracts are used to determine the total l-asparaginase activity. Therefore, this interference does not allow a reliable comparison of results [58].

The l-asparaginase activity values ranged from 0 to 2.29 U g^−1^. The highest values of l-asparaginase activity (>0.5 U g^−1^) were obtained by filamentous fungi isolated from the soil (isolates DCFS6, DCFS10, RCFT14, DCFS1, 2DCSS6 and 2DSST1), whereas all endophytes produced the lowest values of enzymatic activity (<0.5 U g^−1^). The isolates that presented the highest l-asparaginase activity values obtained by quantitative screening after submerged fermentation corroborated with the high zone indices obtained by semi-quantitative screening in solid media and were screened for glutaminase activity.

### 3.3. Glutaminase Screening Using the Quantitative Method

**Figure 3:**
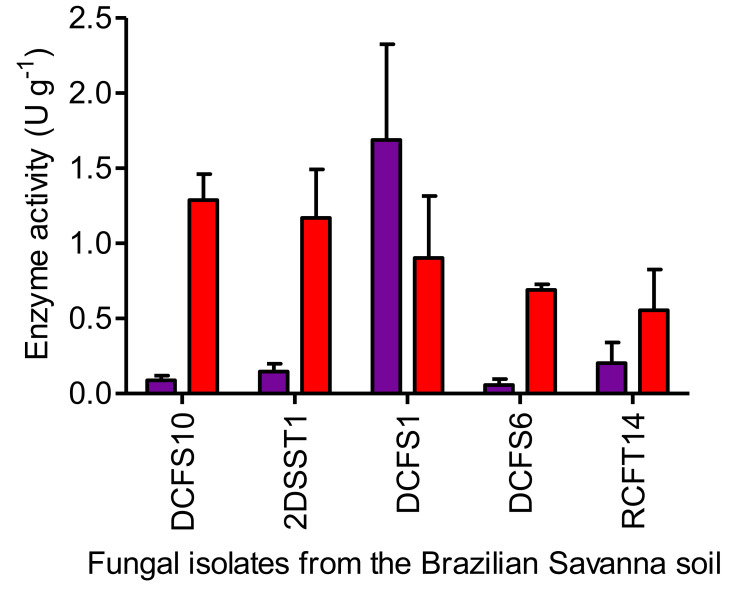
Distribution of enzyme activity quantified in the cells of fungal isolates from the Brazilian Savanna soil grown under submerged fermentation. Results are presented as mean ± standard deviation of enzyme activity. Red bar: l-asparaginase; purple bar: glutaminase.

There was no significant difference between the l-asparaginase and glutaminase activity levels by *Penicillium* sp. RCFT14 (*p* = 0.0821) and *Aspergillus* sp. DCFS1 (*p* = 0.3429), the latter having glutaminase activity higher than l-asparaginase activity (Figure 3). On the other hand, the values of l-asparaginase activity from isolates *Fusarium* sp. DCFS10 (*p* = 0.0022), *Penicillium* sp. 2DSST1 (*p* = 0.0224) and DCFS6 (*p* = 0.0056) were significantly higher than its glutaminase activity; therefore, *Fusarium* sp. DCFS10 and *Penicillium* sp. 2DSST1 were selected for further experiments due to the highest values of l-asparaginase activity with the lowest values of glutaminase activity.

### 3.4. Identification of the Most Promising Cultures

**Figure 4:**
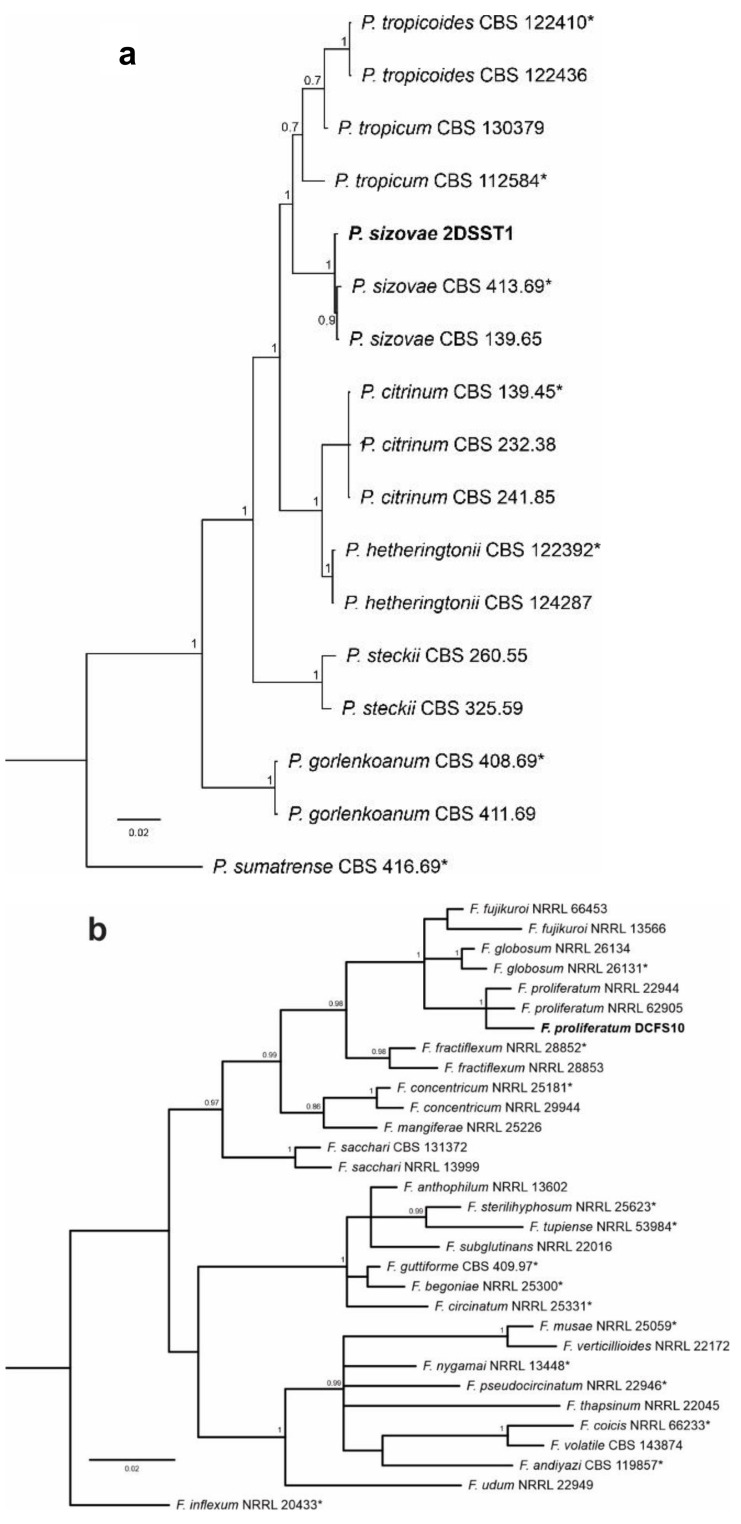
Bayesian phylogenetic tree based on concatenate sequences: (**a**) *its*, *cam*, *tub2*, *rpb*2 of *Penicillium* species section Citrina; (**b**) *tub*2 and *ef*-1*α* of the *Fusarium fujikuroi* species complex. Bayesian posterior probabilities are indicated at the nodes, and the scale bar represents the number of expected changes per site. Ex-type isolates are indicated with an asterisk (*), and the isolates reported here are highlighted in bold.

Upon analysis with the sequences in Genbank and phylogenetic clustering analysis, it was established that the sequences of fungal strains showed a high percentage of identity with *tub*2, *ef*-1*α*, and *its* sequences from *Fusarium* and *tub*2, *cam*, *its* and *rpb*2 sequences from *Penicillium*. The accession numbers in GenBank were MT790711 (*its*), MT815922 (*cam*), MT815923 (*tub*2) and MT815924 (*rpb*2) for *Penicillium* and MT790712 (*its*), MT815925 (*tub*2) and MT815926 (*ef*-1*α*) for *Fusarium*. The same sequences were used in the multigenic identification of species. Based on the multilocus analysis, the isolates *Penicillium* sp. 2DSST1 and *Fusarium* sp. DCFS10 were identified as *Penicillium sizovae* (Figure 4a) and *Fusarium proliferatum* (Figure 4b), respectively.

### 3.5. Screening of Variables for l-Asparaginase Production by Plackett–Burman Design

The first step in process optimization is the screening of important variables, followed by the estimation of optimal levels of these variables [59]. PBD was employed as an efficient screening method to identify the variables that most influence the production of l-asparaginase using as few experimental runs as possible (Table 2). The values of l-asparaginase activities of the isolated *P. sizovae* according to PBD varied between 0 and 3.68 ± 0.14 U mL^−1^. The highest value of l-asparaginase activity was obtained in run number 7, in a culture medium composed of 1% (*w/v*) l-proline, 3% (*w/v*) l-asparagine, 3% (*w/v*) sodium nitrate, 3% (*w/v*) ammonium sulfate, 3% (*w/v*) peptone, 3% (*w/v*) yeast extract, incubated at 32 °C and added with one mycelial disc. The carbon:nitrogen (C:N) ratio of 1.53, the second lowest of all culture media on PBD, shows that the *P. sizovae* strain requires less carbon available in the culture medium to obtain the highest yield of l-asparaginase. The balance of nutrients in a culture medium, especially the C:N ratio, can influence the growth and sporulation of the fungus [60]. It was possible to observe a reduction in the production of fungal biomass in the culture media with the lowest C:N ratios. There was little to no growth of biomass in the culture medium with the lowest C:N ratio, represented by run number 5 according to PBD; therefore, no enzymatic activities were recorded on this run.

The values of l-asparaginase activity of the isolated *F. proliferatum* according to PBD varied between 0.04 ± 0.01 U mL^−1^ and 1.86 ± 0.12 U mL^−1^. The highest values of l-asparaginase activity and specific enzyme activity were obtained in run number 13, in a culture medium composed of 1% (*w/v*) l-proline, 3% (*w/v*) l-asparagine, 3% (*w/v*) ammonium sulfate, 3% (*w/v*) peptone, 1% (*w/v*) glucose, 3% (*w/v*) malt extract, incubated at 32 °C and added with five mycelial discs. The 2.46 C:N ratio shows that the fungus needs approximately two and a half times more carbon than nitrogen in the composition of the culture medium to obtain the highest yield of l-asparaginase.

The results calculated by Protimiza Experimental Design software (Table 4) for l-asparaginase activities (U mL^−1^) and specific enzyme activities (U mg^−1^) obtained by *P. sizovae* show that peptone, yeast extract, urea, l-proline, ammonium sulfate, temperature and l-asparagine are the variables with positive effects on the production of l-asparaginase, that is, they represent an increase in l-asparaginase activities due to an increase in the concentration of variables, while inoculum, sucrose and glucose had negative effects, that is, they represent a decrease in l-asparaginase activities due to an increase in the concentration of variables. Malt extract had a negative effect on the l-asparaginase activity and a positive effect on the specific enzyme activity, that is, the higher the concentration of malt extract, the lower the enzymatic activity and the greater the specific enzyme activity, as it has fewer interfering proteins. Sodium nitrate had a positive effect on the activity of l-asparaginase and a negative effect on the specific enzyme activity, that is, the higher the concentration of sodium nitrate, the greater the enzymatic activity and the lower the specific activity due to the presence of more proteins in the culture medium, probably the increased production of nitrate reductase to hydrolyze the substrate sodium nitrate [61].

The *p* value is used as a tool to check the significance of each of the coefficients, which in turn may indicate the pattern of the interactions between the variables [34]. Peptone, yeast extract, inoculum size, sucrose, glucose, urea, and l-proline are significant variables of the model, while sodium nitrate, l-asparagine and malt extract are not significant variables of the model for both l-asparaginase activity and specific enzyme activity by *P. sizovae*. Ammonium sulfate is significant for l-asparaginase activity but not for specific enzyme activity, while temperature is not significant for l-asparaginase activity but for specific enzyme activity. The highest values of l-asparaginase activity and specific enzyme activity by PBD were obtained by cultivating *P. sizovae* at the lowest level of inoculum in the absence of glucose and sucrose, corroborating the results of significant and negative effects of these variables. For some microorganisms, glucose proved to be a poor carbon source for the production of l-asparaginase, which can also decrease the enzyme’s performance, acting as a repressor when used in greater concentrations [62,63,64,65]. This may be due to catabolic repression caused by glucose and catabolic inhibition of the components involved in lactate transport and by lactate-stimulated l-asparaginase synthesis [66].

Malt extract, temperature, peptone, glucose, inoculum size and l-asparagine are the variables that showed positive effects on the production of l-asparaginase by *F. proliferatum*, whereas ammonium sulfate, sucrose, urea, sodium nitrate and proline showed negative effects. Malt extract was the only significant variable of the model for both l-asparaginase activity and specific enzyme activity. Higher concentrations of carbon sources, such as malt extract and glucose, increased l-asparaginase production by *F. proliferatum*, different from that observed by *P. sizovae*.

### 3.6. Kinetic Parameters of Cellular Growth

**Figure 5:**
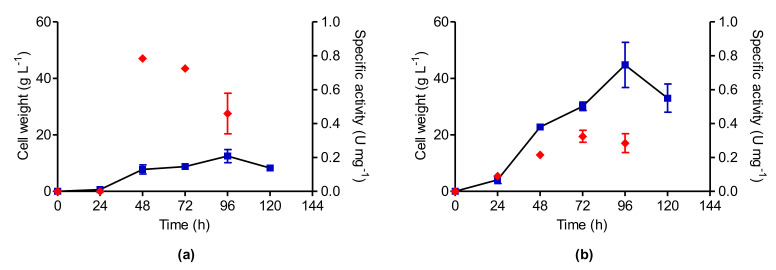
Variation of biomass (■) after 120 h and l-asparaginase specific activity (♦) after 96 h of incubation at 32 °C and 120 rpm: (**a**) *P. sizovae*; (**b**) *F. proliferatum*. Results are presented as mean ± standard deviation.

The lag phase was observed between 0 and 24 h with cell growth lower than 1 g/L and maximum cell growth was observed after 96 h of cultivation for both *P. sizovae* (Figure 5a) and *F. proliferatum* (Figure 5b). *Penicillium sizovae* l-asparaginase was undetectable at 24 h. Maximum l-asparaginase specific activity was observed after 48 h and 72 h of cultivation for *P. sizovae* and *F. proliferatum*, respectively, during the exponential phase, showing that l-asparaginase production is of fungal primary metabolism.

The kinetic parameters of fungal growth and l-asparaginase production were compared when the isolates were cultivated in MCDM before the screening of variables (bPBD) and in the culture medium in which the highest l-asparaginase activity was obtained by PBD (aPBD). The maximum biomass productivity of both isolates was reduced after the screening of culture media variables, which is justified by the reduction of carbon sources in the culture medium, thus reducing fungal biomass. The maximum enzyme productivity was increased 3-fold by *F. proliferatum* and 4-fold by *P. sizovae* after PBD. Specific enzyme yield was increased 4-fold by *F. proliferatum* and 3-fold by *P. sizovae* after the screening of culture media variables. The biomass conversion factor in the enzyme by *F. proliferatum* was increased 4-fold, while by *P. sizovae* it increased 7-fold (Table 5).

### 3.7. Fungal Cell Disruption Mechanical Methods for l-Asparaginase Release

**Figure 6:**
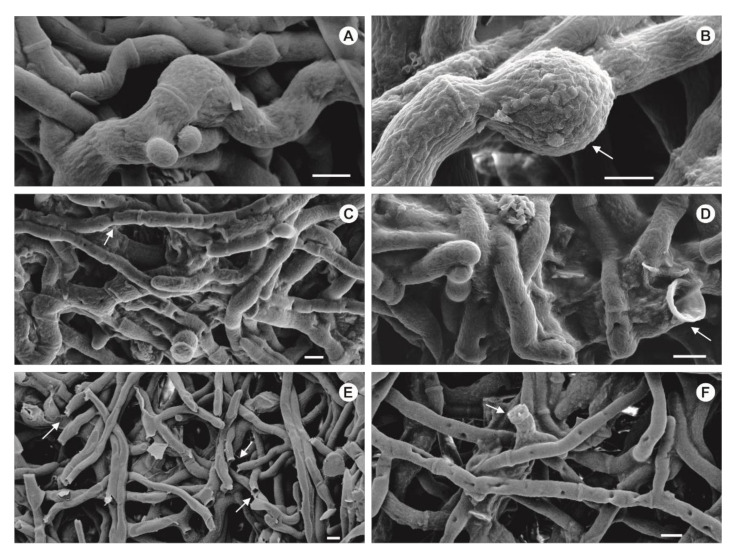
SEM photographs of *Penicillium sizovae*. (**A**,**B**) Hyphal cells and chlamydospore (white arrow) formed in a liquid medium; (**C**,**D**) disruption of hyphae (white arrow) subjected to mechanical extraction by maceration with mortar and pestle; (**E**,**F**) disruption of hyphae (white arrows) and a greater number of holes in the mycelium submitted to physical maceration by sonication. Bars (**A**–**F**) = 2 µm.

Freezing and grinding the fungal biomass was the most efficient method for l-asparaginase release from *P. sizovae*. l-asparaginase activity and specific enzyme activity from *P. sizovae* extracted by the freeze-grinding method (2.35 ± 0.03 U mL^−1^, 0.80 U mg^−1^) were 5-fold and 2-fold, respectively, greater than the values obtained from the sonication method (0.48 ± 0.02 U mL^−1^, 0.36 U mg^−1^), suggesting that the freeze-grinding method promoted fungal cell disruption, thus releasing more l-asparaginase when compared to the sonication method (Figure 6). The highest yield of protein extraction from fungal cell was achieved by mechanical treatment, which was revealed to be the most effective for the disintegration of filamentous fungi cells, specifically *A. fumigatus* and *P. citrinum*, the latter belonging to the same phylogenetic section of *P. sizovae*. Additionally, the freezing and grinding method was the simplest and fastest method used for l-asparaginase release [53].

### 3.8. Scanning Electron Microscopy Analysis

Changes in the mycelium of *P. sizovae* subjected to mechanical methods for fungal cell disruption were observed under SEM and compared with a control sample. It was possible to visualize the terminal chlamydospores formed in the hyphae of the intact biomass used as control (Figure 6A,B). Disrupted hyphae with release of the chlamydospores were visualized in the macerated sample (Figure 6C,D), similar to SEM images of *P. expansum* spores that had been crushed and that exhibited a high degree of hollowness on the spore surface [55]. Disruption of hyphae and a greater number of holes in mycelium were visualized in the sample submitted to physical maceration by sonication (Figure 6E,F). This indicates that disruption is more efficient than the formation of holes in hyphae to release the cellular content. Therefore, it is possible to infer that the maceration method seems to have a deeper damaging effect on fungal cells compared to sonication.

## 4. Conclusions

This study evaluated the biotechnological potential of filamentous fungal isolates from different samples of Brazilian Savanna for the production of l-asparaginase of industrial and pharmaceutical interest. Twenty-one fungal strains obtained from the soil and 18 fungal strains obtained from the leaves of plants from the Brazilian Savanna were screened for enzyme production, out of which two fungi, *P. sizovae* and *F. proliferatum*, obtained from the soil were selected as the greatest l-asparaginase producers with the lowest glutaminase activity. The Plackett–Burman design was used to evaluate the culture medium variables that influence l-asparaginase activity and specific enzyme activity. The highest values of total enzymatic activity were recorded at 3.68 ± 0.14 U mL^−1^ and 1.86 ± 0.12 U mL^−1^ for *P. sizovae* and *F. proliferatum*, respectively. Carbon sources such as glucose, sucrose and malt extract were found to be a repressor of enzyme synthesis by *P. sizovae*. On the other hand, l-asparaginase production by *F. proliferatum* was enhanced with higher levels of carbon sources such as glucose and malt extract and inoculum size. The Plackett–Burman design improved the enzyme productivity, specific enzyme yield and biomass conversion factor in the enzyme of the isolates. Freeze-grinding was proved to be the best mechanical method for the disruption of fungal cells for l-asparaginase release, as it was seen that it promoted the extravasation of cellular content of *P. sizovae* compared to sonicated and control samples. This study shows the upstream processing of l-asparaginase production by fungal species isolated from the Brazilian Savanna soil with low glutaminase activity. The evaluation of nutrient and culture conditions as well as the evaluation of efficient fungal cell disruption methods enhanced the l-asparaginase yield, thus being the most efficient and optimized methods employed in upstream processing for l-asparaginase production by the selected isolates. The discovery of novel l-asparaginase producers such as *P. sizoave* and *F. proliferatum* of eukaryotic origin with low glutaminase activity isolated from a global biodiversity hotspot may lead to safer alternatives for patients who develop treatment-limiting side effects due to their potential to be less immunogenic and thus is an improvement in ALL therapy worldwide.

## Figures and Tables

**Table 1:** Values of the variables employed in Plackett–Burman experimental design.

Variables	Units	−1	0	+1
l-proline (X_1_)	% (*w/v*)	1	2	3
l-asparagine (X_2_)	% (*w/v*)	1	2	3
Urea (X_3_)	% (*w/v*)	0	0.5	1
Sodium nitrate (X_4_)	% (*w/v*)	0	1.5	3
Ammonium sulfate (X_5_)	% (*w/v*)	0	1.5	3
Peptone (X_6_)	% (*w/v*)	0	1.5	3
Yeast extract (X_7_)	% (*w/v*)	0	1.5	3
Glucose (X_8_)	% (*w/v*)	0	0.5	1
Sucrose (X_9_)	% (*w/v*)	0	0.5	1
Malt extract (X_10_)	% (*w/v*)	0	1.5	3
Temperature (X_11_)	°C	28	30	32
Inoculum size (X_12_)	Units *	1	3	5

* Units of mycelial disc (8 mm in diameter).

**Table 2:** Plackett–Burman experimental design matrix for the screening of variables for l-asparaginase activity (Y_1_) and specific enzyme activity (Y_2_) by *P. sizovae* and *F. proliferatum*.

Run	X_1_ (%)	X_2_ (%)	X_3_ (%)	X_4_ (%)	X_5_ (%)	X_6_ (%)	X_7_ (%)	X_8_ (%)	X_9_ (%)	X_10_ (%)	X_11_ (°C)	X_12_ (units)	C:N	*P. sizovae*		*F. proliferatum*	
														Y_1_ (U mL^−1^)	Y_2_ (U mg^−1^)	Y_1_ (U mL^−1^)	Y_2_ (U mg^−1^)
1	3	1	0	0	3	0	0	1	1	0	32	1	2.27	0.28 ± 0.03	0.09	0.08 ± 0.01	0.03
2	3	3	0	0	0	3	0	0	1	3	28	5	3.91	0.66 ± 0.02	0.21	0.22 ± 0.01	0.09
3	3	3	1	0	0	0	3	0	0	3	32	1	2.82	2.46 ± 0.10	1.03	0.46 ± 0.04	0.13
4	3	3	1	3	0	0	0	1	0	0	32	5	1.66	0.48 ± 0.05	0.20	0.19 ± 0.01	0.07
5	1	3	1	3	3	0	0	0	1	0	28	5	0.95	ND	ND	0.04 ± 0.01	0.01
6	3	1	1	3	3	3	0	0	0	3	28	1	1.76	2.96 ± 0.08	0.81	0.23 ± 0.00	0.06
7	1	3	0	3	3	3	3	0	0	0	32	1	1.53	3.68 ± 0.14	0.87	0.14 ± 0.01	0.03
8	3	1	1	0	3	3	3	1	0	0	28	5	2.05	2.06 ± 0.03	0.65	0.22 ± 0.00	0.05
9	3	3	0	3	0	3	3	1	1	0	28	1	2.65	1.91 ± 0.13	0.29	0.39 ± 0.03	0.10
10	1	3	1	0	3	0	3	1	1	3	28	1	2.23	1.08 ± 0.01	0.31	0.45 ± 0.01	0.11
11	1	1	1	3	0	3	0	1	1	3	32	1	2.54	1.24 ± 0.04	0.31	0.77 ± 0.01	0.19
12	3	1	0	3	3	0	3	0	1	3	32	5	2.26	0.90 ± 0.04	0.26	0.47 ± 0.01	0.14
13	1	3	0	0	3	3	0	1	0	3	32	5	2.46	0.71 ± 0.01	0.22	1.86 ± 0.12	0.44
14	1	1	1	0	0	3	3	0	1	0	32	5	2.56	1.74 ± 0.09	0.46	0.83 ± 0.01	0.20
15	1	1	0	3	0	0	3	1	0	3	28	5	3.02	0.33 ± 0.00	0.08	0.62 ± 0.02	0.16
16	1	1	0	0	0	0	0	0	0	0	28	1	2.65	0.27	0.07	0.17 ± 0.00	0.05
17	2	2	0.5	1.5	1.5	1.5	1.5	0.5	0.5	1.5	30	3	2.21	1.82 ± 0.09	0.55	0.15 ± 0.00	0.06
18	2	2	0.5	1.5	1.5	1.5	1.5	0.5	0.5	1.5	30	3	2.21	1.33 ± 0.05	0.44	0.12 ± 0.00	0.03
19	2	2	0.5	1.5	1.5	1.5	1.5	0.5	0.5	1.5	30	3	2.21	1.18 ± 0.01	0.39	0.18 ± 0.00	0.05

X_1_: l-proline; X_2_: l-asparagine; X_3_: urea; X_4_: sodium nitrate; X_5_: ammonium sulfate; X_6_: peptone; X_7_: yeast extract; X_8_: glucose; X_9_: sucrose; X_10_: malt extract; X_11_: temperature; X_12_: inoculum size. Results are given as mean ± standard deviation. ND: not detectable.

**Table 3:** Zone index values for the isolates obtained from Brazilian Savanna samples using phenol red as indicator representing their extent of crude l-asparaginase production after 48 h of incubation.

Species	Isolate	Sample Nature	Zone Index
*Aspergillus* sp.	DCFS1	Soil	4.55
*Penicillium* sp.	DCFS6	Soil	4.00
*Penicillium* sp.	RCFS24	Soil	3.85
*Aspergillus terreus*	2DCSS6	Soil	3.64
*Penicillium* sp.	2DSST1	Soil	3.64
*Penicillium* sp.	2DMGSE2	Soil	3.64
*Penicillium* sp.	RCFS6	Soil	3.13
*Penicillium* sp.	2DSST10	Soil	3.00
NI	CAG2	Plant (*Eugenia dysenterica*)	2.81
*Penicillium* sp.	RCFT14	Soil	2.67
*Fusarium* sp.	DCFS10	Soil	2.50
*Penicillium* sp.	DCFF2	Soil	2.50
NI	EP03	Plant (*Eriotheca pubescens*)	2.22
NI	EP01	Plant (*Eriotheca pubescens*)	2.20
NI	PEQ02	Plant (*Caryocar brasiliense*)	1.96
*Penicillium* sp.	2DSSSE1	Soil	1.86
*Penicillium* sp.	DCFF4	Soil	1.79
NI	IPE03	Plant (*Tabebuia ochracea*)	1.74
NI	EP04	Plant (*Eriotheca pubescens*)	1.60
*Penicillium* sp.	2RCSS1	Soil	1.00
*Penicillium* sp.	DCFT5	Soil	1.00
NI	IPE05	Plant (*Tabebuia ochracea*)	1.00
NI	PT02	Plant (*Pouteria torta*)	1.00
*Aspergillus niger*	RCFS17	Soil	1.00
*Penicillium* sp.	DCFT2	Soil	0.87
*Penicillium* sp.	RCFS7	Soil	0.83
*Trichoderma* sp.	RCFS21	Soil	0.38
NI	BR	Plant (*Sapindus saponaria*)	-
NI	CAG	Plant (*Eugenia dysenterica*)	-
NI	CAG1	Plant (*Eugenia dysenterica*)	-
NI	CAG3	Plant (*Eugenia dysenterica*)	-
NI	CAM01	Plant (*Calophyllum brasiliense*)	-
NI	CB02	Plant (*Calophyllum brasiliense*)	-
*Aspergillus* sp.	DCFS9	Soil	-
NI	GOI03	Plant (*Psidium guajava* L.*)*	-
NI	IPE02	Plant (*Tabebuia ochracea*)	-
NI	OH01	Plant (*Ouratea hexasperma*)	-
NI	OH03	Plant (*Ouratea hexasperma*)	-
*Fusarium* sp.	RCFS3	Soil	-

NI: not identified; ′-′ indicates no detection of color change.

**Table 4:** Effects and significance of variables for l-asparaginase activity and specific enzyme activity by *P. sizovae* and *F. proliferatum* from the results of Plackett–Burman experimental design.

Name	*P. sizovae*						*F. proliferatum*					
	l-Asparaginase Activity			Specific Activity			l-asparaginase Activity			Specific Activity		
	Effect	Calculated t	*p-*Value	Effect	Calculated t	*p-*Value	Effect	Calculated t	*p-*Value	Effect	Calculated t	*p-*Value
Mean	1.30	17.77	0.0000	0.37	16.27	0.0000	0.45	4.96	0.0043	0.12	5.73	0.0023
l-proline (x_1_)	0.33	2.27	0.0721	0.15	3.39	0.0195	−0.33	−1.81	0.1293	−0.07	−1.60	0.1703
l-asparagine (x_2_)	0.15	1.04	0.3456	0.05	1.11	0.3173	0.04	0.25	0.8132	0.01	0.31	0.7706
Urea (x_3_)	0.41	2.80	0.0379	0.21	4.66	0.0055	−0.10	−0.54	0.6141	−0.03	−0.68	0.5283
Sodium nitrate (x_4_)	0.28	1.91	0.1142	−0.03	−0.61	0.5681	−0.18	−1.00	0.3628	−0.04	−1.05	0.3431
Ammonium sulfate (x_5_)	0.32	2.19	0.0798	0.07	1.55	0.1808	−0.02	−0.11	0.9191	−0.02	−0.37	0.7269
Peptone (x_6_)	1.15	7.84	0.0005	0.22	4.94	0.0043	0.27	1.52	0.1887	0.06	1.42	0.2159
Yeast extract (x_7_)	0.95	6.47	0.0013	0.26	5.66	0.0024	0.00	0.00	0.9985	0.00	−0.06	0.9533
Glucose (x_8_)	−0.57	−3.92	0.0112	−0.19	−4.33	0.0075	0.25	1.41	0.2188	0.06	1.35	0.2335
Sucrose (x_9_)	−0.64	−4.40	0.0070	−0.25	−5.55	0.0026	−0.08	−0.45	0.6705	−0.01	−0.37	0.7269
Malt extract (x_10_)	−0.01	−0.07	0.9459	0.08	1.67	0.1567	0.37	2.08	0.0922	0.10	2.40	0.0615
Temperature (x_11_)	0.28	1.89	0.1176	0.13	2.83	0.0366	0.31	1.72	0.1468	0.08	1.85	0.1240
Inoculum (x_12_)	−0.88	−5.99	0.0019	−0.21	−4.72	0.0052	0.22	1.21	0.2821	0.06	1.42	0.2159

**Table 5:** Kinetic parameters of growth and l-asparaginase production by *P. sizovae* and *F. proliferatum*.

Kinetic Parameter	Symbol	Units	*P. sizovae*		*F. proliferatum*	
			bPBD	aPBD	bPBD	aPBD
Maximum biomass productivity	P_X,max_	g(X)/L·h	1.31	0.16	0.70	0.48
Maximum enzyme productivity	P_E,max_	U/L·h	12.85	56.90	11.36	33.68
Specific growth rate	µ_max_	h^−1^	0.08	0.11	0.08	0.07
Specific enzyme yield	µ_E,max_	U/g(X)·h	1.94	7.30	1.83	8.22
Biomass conversion factor in enzyme	Y_E/X_	U/g(U)	46.63	350.16	43.98	197.17

bPBD: before Plackett–Burman design; aPBD: after Plackett–Burman design.

## Data Availability

Not applicable.
